# Quasi-Periodic Dendritic Metasurface for Integral Operation in Visible Light

**DOI:** 10.3390/molecules25071664

**Published:** 2020-04-04

**Authors:** Huan Chen, Di An, Xiaopeng Zhao

**Affiliations:** Smart Materials Laboratory, Department of Applied Physics, Northwestern Polytechnical University, Xi’an 710129, China; chenhuan151@mail.nwpu.edu.cn (H.C.); di_an@163.com (D.A.)

**Keywords:** dendritic metasurface, integral operation, electrochemical deposition

## Abstract

A reflective metasurface model composed of silver dendritic units is designed in this study. The integral property of this metasurface, which consists of an upper layer of dendritic structures, a silica spacer, and a bottom silver substrate was demonstrated at visible wavelengths. The simulation results revealed that the metasurface can perform integral operation in the yellow and red bands; this can be easily generalized to the infrared and communication bands by scaling the transverse dimensions of this metasurface. A dendritic metasurface sample responding to red light was prepared via the bottom-up electrochemical deposition method. The integral operation property of the sample was verified experimentally. This dendritic metasurface, which can perform integral operation in visible light, can be used for big data processing technology, real-time signal processing, and beam shaping, and provides a new method for miniaturized and integrated all-optical signal processing systems.

## 1. Introduction

Traditional analog computers primarily perform calculations using variable physical quantities of mechanical, electronic, or hybrid forms [1,2]. These forms result in some inherent defects, such as large size and slow response, which significantly limit the development of these computers, whereas digital computing technology has been developed to a great extent [3,4]. Although optical analog computers can theoretically overcome these limitations, many problems remain in current optical transistors and digital logic circuits [5]. The manipulation of light waves using conventional lens-based optical signal processors relies on the gradual accumulation of phase delays, making these devices inherently deficient in the bulk and diffraction limits [6], among others.

In recent years, there has been renewed interest in the scientific community toward optical analog computing with the rapid development of metamaterials, because of their advantageous characteristics of fast speed, high timeliness, high data fidelity, and parallel processing, which are superior to those of digital computing. In 2014, Silva et al. conceptualized “computing metamaterials” and designed a metasurface model that could perform various mathematical operations in the near-infrared band [7]. Metasurfaces are 2D metamaterials that arbitrarily manipulate the phase, amplitude, or polarization of an impinging light using subwavelength structures [8,9,10]. Metasurfaces have gained the attention of many researchers because of their simple fabrication process and low loss [9,11,12,13,14,15,16,17,18,19,20,21,22]. Motivated by the work on metamaterial-based analog computing [7], researchers have proposed various mathematical metasurfaces working in the near-infrared or infrared bands [16,22,23,24,25,26,27,28,29]. Optical processors consisting of metasurfaces are significantly thinner than conventional lens-based signal processors, thereby enabling the possibility of developing miniaturized and integrated ultrafast all-optical signal processing systems. However, the existing mathematical metasurfaces comprise periodic structure units, and the size, shape, or material composition of each nanostructure unit should be accurately controlled to realize mathematical operation. For example, in 2016, Chizari et al. [28] proposed a mathematical metasurface based on an engineered gradient dielectric meta-reflect array and simulated its mathematical operations, including differential and integral operations in the infrared band. Thus, fabricating these metasurface samples remains challenging, even in the infrared band. Mathematical metasurfaces operating in the visible light band have rarely been reported because of the smaller size of their structural units. A small number of metasurface samples have been prepared by etching technology, which is expensive, complicated, and not easily popularized [22].

Integral metasurfaces have attracted the attention of many researchers, but the realization of integral operation in the visible band has been a difficult problem due to the difficulty of sample preparation. Previously, we have presented an easy-to-prepare reflective dendritic metasurface that could perform differential operations at visible wavelengths to address the limitations in preparing visible light metasurfaces [30]. Simulations indicated that this metasurface can realize differential operation in red, yellow, and green bands. Metasurface samples that respond to green and red bands were prepared using the simple electrochemical deposition method, and their differential operation properties were experimentally demonstrated. Here, we further present an integral metasurface comprising of dendritic structures [31,32], considering the existing preparation process devised by our group [33,34,35]. The proposed metasurface consists of upper silver dendritic structures, an intermediate silica spacer, and an Ag substrate. By utilizing the electric and magnetic resonances, similar to the Fabry–Perot resonance [16,36], of the dendritic structure and multiple reflections of light waves within the dielectric spacer, the amplitude and phase profiles of the reflected co-polarized light can be fully manipulated by varying the shapes of the dendritic structures. Simulations were performed to validate the integral operation property of the metasurface in the yellow and red bands. An Ag dendritic metasurface sample is prepared via the electrochemical deposition method. An experiment revealed that the sample can perform integral operation in the red band. An integration processor based on this metasurface can be used for real-time optical signal processing and optical communication and could be utilized for shaping optical pulses [37]. Moreover, the optical integrators can potentially be used as the fundamental building blocks of all-optical signal processing systems.

## 2. Design of Integral Metasurfaces

### 2.1. Design Principle

First, we review the design idea of the integral metasurface. In a linear invariant space, the relationship between any input function E_in_(x, y) and the corresponding output function E_re_(x, y) can be expressed by the following linear convolution relation [7]:

(1) $$E_{re}(x,y)=h(x,y)\times E_{in}(x,y)$$

where h(x, y) represents the transfer function in the space. In the Fourier space, Equation (1) is transformed into:

(2) $$E_{re}(k_{x},k_{y})=H(k_{x},k_{y})E_{in}(k_{x},k_{y})$$

where E_re_(k_x_, k_y_) = FT{E_re_(x, y)}, H(k_x_, k_y_) = FT{h(x, y)}, E_in_(k_x_, k_y_) = FT{E_in_(x, y)}, FT represents the Fourier transform, and (k_x_, k_y_) denotes the 2D variables in the Fourier space.

**Figure 1:**
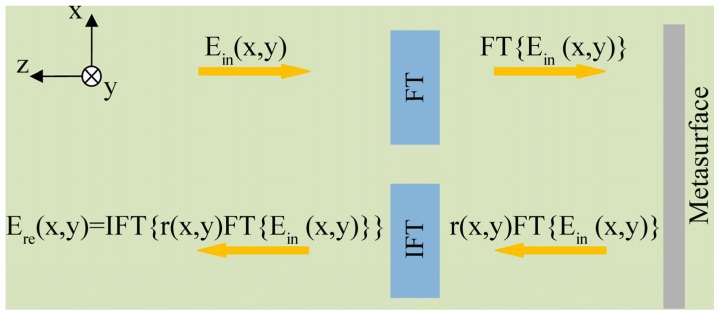
A schematic showing mathematical operation on a reflective metasurface. The system consists of an FT module, an inverse FT (IFT) module, and a metasurface with a position-dependent reflection coefficient r(x, y).

As shown in Figure 1, E_in_(x, y) represents the incident linearly polarized light; E_re_(x, y) denotes the linearly co-polarized light reflected by the metasurface; H(k_x_, k_y_) is the transfer function associated with the desired mathematical operation, which is equal to the position-dependent reflection coefficient r(x, y) of the metasurface (here, the real-space coordinates (x, y) act as Fourier-space variables (k_x_, k_y_)). Thus, Equation (2) can be expressed as follows:

(3) $$E_{re}(x,y)=IFT\left\{r(x,y)FT\left\{E_{in}(x,y)\right\}\right\},$$

for the 1D condition,

(4) $$E_{re}(x)=IFT\left\{r(x)FT\left\{E_{in}(x)\right\}\right\}.$$

Fourier transform (FT) can be achieved by an ordinary lens or compact gradient-index lens [7,28,38] or even focusing metasurfaces [39]; however, natural materials cannot be used to implement inverse FT (IFT). Therefore, based on the FT law, $FT\left\{FT\left\{E_{re}(x)\right\}\right\}=E_{re}(-x)$, a mirror image E_re_(−x) of the desired output E_re_(x) is obtained.

In terms of Equation (4), r(x) must imitate the integral operator to perform the integral operation for E_in_(x) in the Fourier space. Given that the integral operator is transformed to (ik_x_)^−1^ in the Fourier space, the reflection coefficient amplitude of the integral metasurface is as follows [16]:

(5) $$r_{\mathrm{int}}(x)=\{\begin{array}{c} r_{m},\left|x\right|<d\\ r_{m}d/x,\left|x\right|\geq d \end{array}$$

where −l≤x≤l, 2*l* is the size of the metasurface in the x-direction, $r_{m}\leq 1$ indicates the maximum reflection coefficient amplitude, and d≪l defines a region near the center of the metasurface with a constant reflection amplitude r_m_.

### 2.2. Design of Ag Dendritic Metasurface

**Figure 2:**
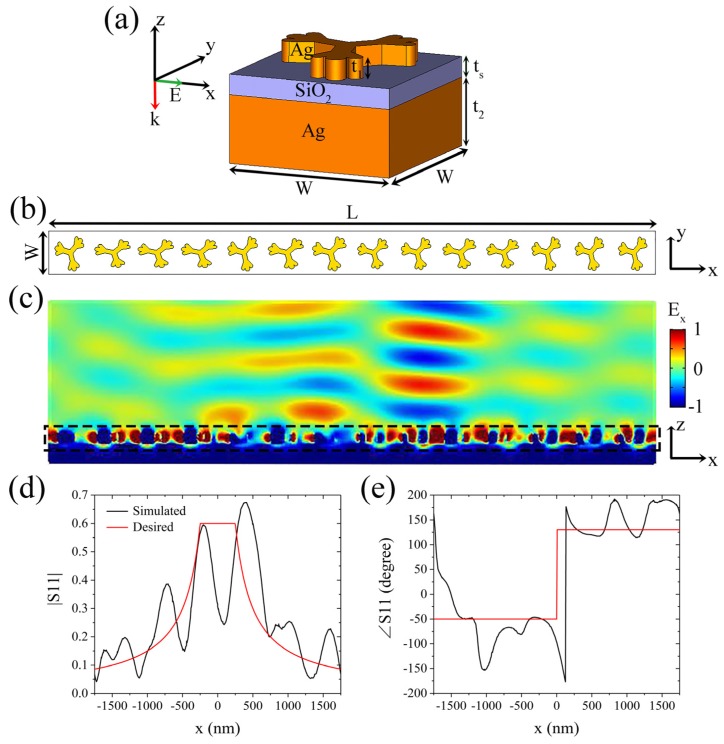
Design and simulation of dendritic metasurface integrator. (**a**) 3D view of the unit in the integral metasurface. t_1_ = 30 nm, t_s_ = 30 nm, t_2_ = 100 nm, and W = 250 nm, and the incident x-polarized light propagates normally through the metasurface along the –z-axis direction. (**b**) Integral metasurface with 14 different shapes of Ag dendritic units. L = 2*l* = 14W = 3500 nm. (**c**) Reflected co-polarized electric field profile obtained when yellow light of wavelength 586 nm is incident on the metasurface. (**d**) and (**e**) show the simulated (black line) and theoretical (red line) position-dependent reflection coefficient amplitude and phase, respectively.

A unit composed of an upper Ag dendritic structure, SiO_2_ spacer, and bottom Ag film is designed, as shown in Figure 2a. It is worth noting that the designed dendritic structure in this paper is a fractal-like structure similar to the second-order Cayley trees [40,41]. In addition, the dendritic structure has been called an artificial molecule [42]. The periodicity of the unit is W = 250 nm, and the thicknesses of the Ag dendritic structure, SiO_2_ dielectric, and Ag film are respectively t_1_ = 30 nm, t_s_ = 30 nm, and t_2_ = 100 nm (Ag film of this thickness can totally reflect the incident light). The permittivity of SiO_2_ is 2.1, and the permittivity of Ag is set as the actual Drude model value [43]. Extensive simulations were performed [30], whose results show that changing the shape of the Ag dendritic structure in the resonant band allows for abrupt arbitrary changes in the phase and amplitude of the co-polarized light reflected by the unit. A metasurface with 14 different shapes of Ag dendritic units is designed to realize Equation (5), as shown in Figure 2b. Further, L = 2*l* = 14W is the dimension of the metasurface in the x-direction. Considering the intrinsic loss of material and other factors, the maximum achievable reflection amplitude is set to be lower than 1. Here, we select r_m_ = 0.6 and d = 250 nm based on a previous simulation [30].

## 3. Simulation and Experiment for the Integral Property of the Dendritic Metasurface

### 3.1. Simulation

The position-dependent reflection coefficient of the Ag dendritic metasurface illustrated in Figure 2b is simulated using the finite element software COMSOL Multiphysics 5.2, and the boundaries of the metasurface in the x and y directions are set to the Floquet periodic boundary conditions. A linearly x-polarized light of wavelength 586 nm is incident perpendicular to the metasurface along the –z-axis. Figure 2c shows the reflected co-polarized electric field E_x_ of the metasurface, which is consistent with the desired output. The simulated position-dependent reflection coefficient amplitude and phase of the metasurface are shown in Figure 2d,e, respectively. In Figure 2d, the comparison of the simulated amplitude curve (black line) and the theoretical prediction (red line) indicates that the maximum deviation is approximately 0.18, except for the area near the center of the metasurface, which is slightly larger than that in the published paper [28]. In Figure 2e, compared with the theoretical prediction, the simulated phase curve has a maximum deviation of 61°, except for the drastic phase change near the center and the boundary of the metasurface, which is larger than that in the published paper [28]. Although these deviations are larger than those in the published paper, they are acceptable. Thus, we demonstrate that the Ag dendritic metasurface has the property of integral operation in the yellow band.

**Figure 3:**
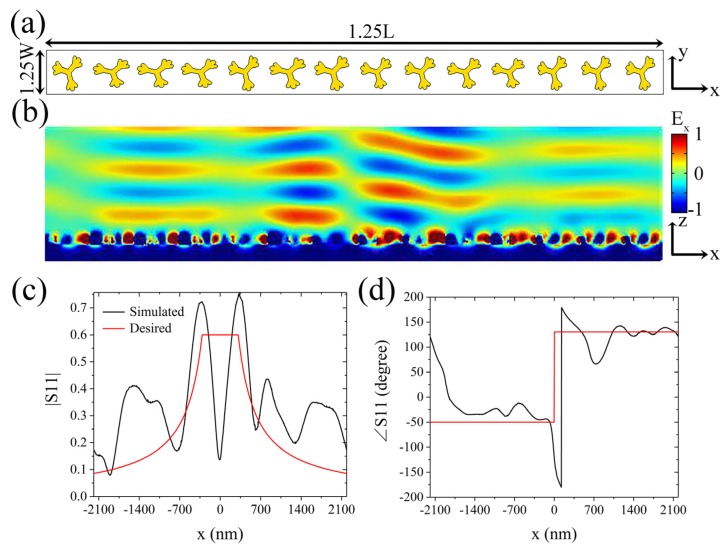
Simulation of the integral metasurface in the red band. (**a**) Metasurface model with a transverse magnification of 1.25 times. (**b**) Reflected co-polarized electric field profile obtained when red light of wavelength 632 nm is incident on the metasurface. (**c**) and (**d**) show the simulated (black line) and theoretical (red line) position-dependent reflection coefficient amplitude and phase, respectively.

Then, we attempt to change the operating wavelength by scaling the transverse dimensions (i.e., the dimensions in the xy plane) of the metasurface presented in Figure 2b. Figure 3a shows a metasurface in which all the transverse dimensions are enlarged by 1.25 times compared with those in the metasurface displayed in Figure 2b. Here, d is set to 312.5 nm. The reflected electric field profile of the metasurface, when the wavelength of the incident light is 632 nm, is shown in Figure 3b, which is also consistent with the desired output electric field. The position-dependent reflection coefficient is shown in Figure 3c,d. Compared with the theoretical prediction, the maximum deviation of the simulated amplitude curve, excluding the region near the center of the metasurface, is 0.29, which is larger than that in the published paper [28]. Compared with the theoretical prediction, the corresponding phase curve has a maximum deviation of 38.2°, except for the phase change near the center and the boundary of the metasurface, which is slightly larger than that in the published paper [28]. These results are consistent with the theoretical predictions; thus, the Ag dendritic metasurface can perform integral operation in the red band.

Consequently, we first propose and verify by simulation that the Ag dendritic metasurface can perform integral operation in the yellow and red bands. According to our previous research [30], the three-layer structural unit designed in this way has high absorption in the resonance band, which makes the reflection very low; however, when the shape of the upper dendritic structure is different, the resonance band of the structural unit is also different. Therefore, when a metasurface is composed of a plurality of dendritic structures of different shapes, irradiation of incident light with a certain wavelength on the metasurface will cause some structural units to resonate, thereby generating low reflections; while a small number of structural units will not resonate and produce high reflections, as shown in Figure 2d and Figure 3c. The simulation results shown in Figure 2d and Figure 3c indicate that the working wavelength of the dendritic metasurface is red-shifted with the proportional enlargement of the transverse dimensions of the metasurface; moreover, the maximum reflection coefficient amplitude increases because of the lower loss with the larger structures in the metasurface. The maximum deviation of the amplitude increases slightly with the increase in the transverse dimensions of the metasurface, which arises from the significant difference between the morphology of the dendritic units and that of the other periodic structures [16,28]. The reflected light waves destructively interfere at x = 0 because in the designed metasurface, the phase difference between the abrupt phases produced by the two structural units on both sides of x = 0 is approximately 180°, and the intensity of the light waves they reflect is nearly equal. Therefore, at x = 0, both the simulated curves in Figure 2d and Figure 3c exhibit noticeable troughs. Additionally, these results indicate that the operating wavelength of the metasurface model shown in Figure 2a can be easily adjusted by scaling its transverse dimensions. Thus, the integral property of the metasurface can be extended to the infrared and communication bands.

### 3.2. Preparation of Ag Dendritic Metasurface Sample

**Figure 4:**
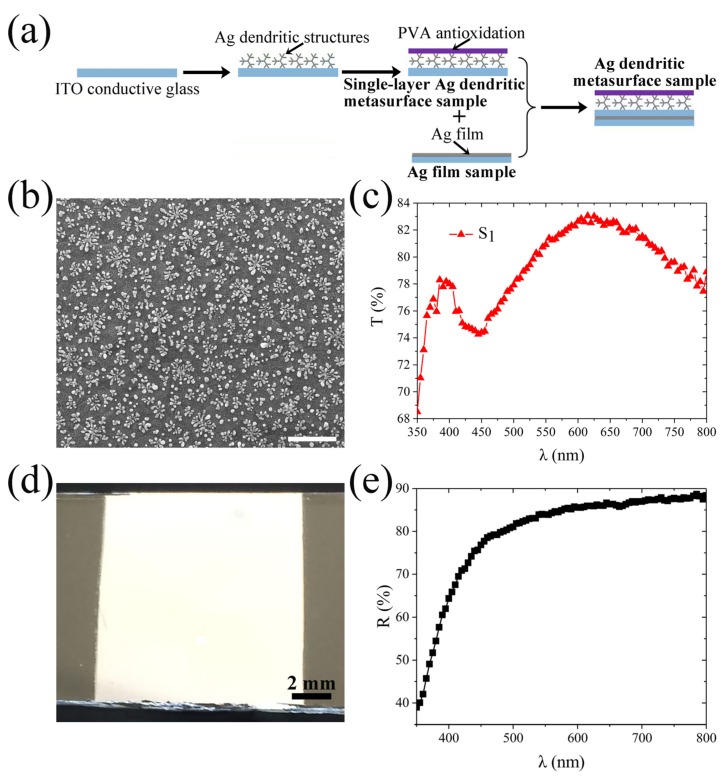
Ag dendritic metasurface sample. (**a**) Schematic of the assembly process of Ag dendritic metasurface sample. (**b**) SEM image of the prepared single-layer Ag dendritic metasurface sample without the polyvinyl alcohol (PVA) coating. Scale bar: 500 nm. (**c**) Transmission spectrum of the prepared single-layer Ag dendritic metasurface sample. (**d**) Optical photo of the prepared Ag film sample under natural light. (**e**) Reflection spectrum of the prepared Ag film sample.

The Ag dendritic metasurface sample is prepared by adopting the bottom-up electrochemical deposition method proposed by our group [44]. The sample is assembled from a single-layer Ag dendritic metasurface sample and an Ag film sample, which are prepared through the electrochemical deposition method. The preparation process is illustrated in Figure 4a. The prepared single-layer Ag dendritic metasurface sample has an area of 13 mm × 10 mm [44]. In Figure 4b, the scanning electron microscope (SEM) image indicates that the size of the Ag dendritic structures varies from 200 nm to 300 nm. Therefore, there are approximately 10^8^ dendritic structures of various shapes in the prepared sample. These dendritic structures exhibit a quasi-periodic distribution on the indium tin oxide (ITO) conductive glass. These dendritic structures must contain the designed dendritic structures in the simulation for this metasurface sample to be used to verify the integral property of the previously designed dendritic metasurface. The transmission spectrum of the single-layer Ag dendritic metasurface sample S_1_ is shown in Figure 4c. A high transmission resonance peak is observed at 620 nm in addition to the intrinsic resonance peak of Ag at approximately 400 nm, which indicates that the response band of this sample is the red band. Therefore, sample S_1_ can represent the metasurface model shown in Figure 3a. Furthermore, the response band of the metasurface sample can be regulated by changing the deposition conditions, such as deposition voltage, deposition time, and experimental temperature [44]. As much of our previously published work [30,32,42,44] has given in detail the sample preparation process and various characterizations of the structures in the sample, we do not present a more detailed sample characterization here.

The preparation process of an Ag film sample of the same area is similar to that of preparing a single-layer Ag dendritic metasurface sample [30]. An optical photograph of the prepared Ag film sample is presented in Figure 4d, which shows that the Ag film is dense and bright. The reflection spectrum in Figure 4e shows that the reflectance of the Ag film is approximately 86% at 632 nm, which nearly satisfies the requirement that the Ag film should reflect all the incident light.

The Ag film sample is placed in close contact with the dendritic metasurface sample S_1_ to obtain the metasurface sample S_2_ for the final test, as shown in Figure 4a. Therefore, the Ag dendritic metasurface sample is in fact composed of an uppermost polyvinyl alcohol (PVA) antioxidation layer, Ag dendritic structure layer, ITO conductive glass layer, Ag film layer, and ITO conductive glass substrate. For the transmission spectrum of the single-layer dendritic sample in Figure 4c, the transmission peak in the visible band proves that the distribution of dendritic structures in the sample is statistically quasi-periodic [30,32]. For the reflection spectrum of the silver film sample in Figure 4e, the high reflectance in the visible band indicates that the prepared silver film is a good reflective substrate [28,30]. Therefore, the reflection and transmission data graphs present here are a good illustration of our idea. It is worth noting that the entire preparation process of the metasurface sample is simple and mature, and the cost is relatively lower than that of the etching technique, which is advantageous for a wide application of the metasurface.

### 3.3. Reflectivity Measurement of Dendritic Metasurface

**Figure 5:**
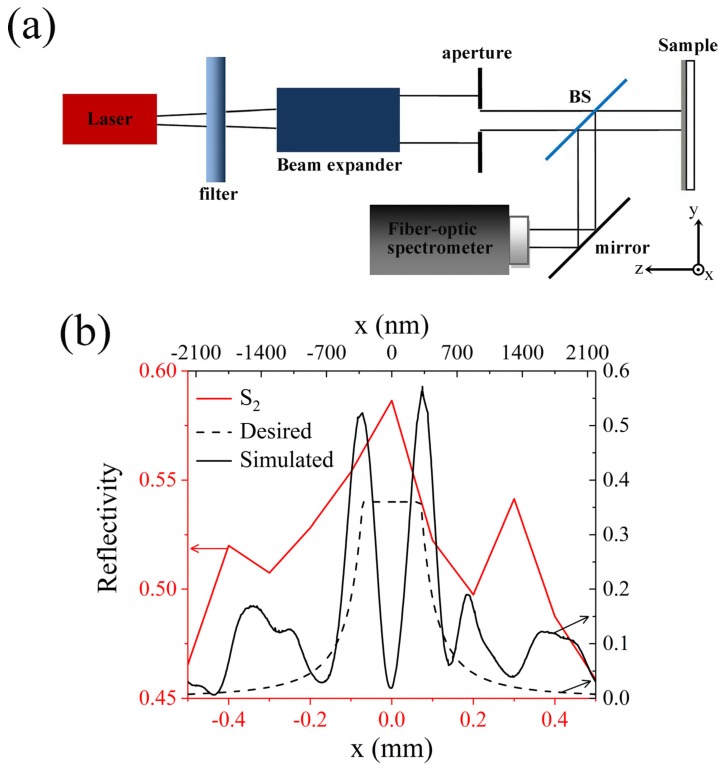
Experiment on the integral property of the dendritic metasurface sample. (**a**) Schematic of the experimental device for measuring the reflected light intensity of the sample. (**b**) Distribution of normalized co-polarized reflected light intensity (i.e., reflectivity) of sample S_2_ along the x-axis.

The principle for testing the position-dependent reflectivity of the Ag dendritic metasurface sample is shown in Figure 5a. After the x-polarized light beam emitted from the laser passes through the filter, its intensity is regulated to an appropriate level. Then, the spot diameter of the beam is enlarged through the beam expander. The spot diameter of the enlarged beam is adjusted to the appropriate size after the beam passes through the circular aperture. Then, a part of the beam penetrates the beam splitter (BS) and vertically irradiates the sample. The other part of the light reflected from the sample is reflected by the BS toward the mirror. Finally, the light reflected by the mirror is received by the probe connected with the fiber-optic spectrometer. During the measurement, the intensity of the reflected spot at various positions in the direction is determined by moving the probe along the x-direction and through the center of the spot. An He-Ne laser of wavelength 632.5 nm is used as the light source in this study.

The reflected light intensities of the Ag dendritic metasurface sample S_2_ and Ag film sample along the x-axis are separately measured using the device shown in Figure 5a. Thereafter, the reflected light intensity of sample S_2_ is normalized by the reflected light intensity of the Ag film sample; the normalized reflected light intensity distribution (i.e., position-dependent reflectivity) along the x-axis is depicted in Figure 5b. The area of the sample is much larger than that of the incident spot; thus, the actual region of the sample measured is the region covered by the spot. Figure 5b indicates that the change in the trend of the measured curve is consistent with that of the theoretical prediction. In contrast, the reflectivity in the portion near the edge of the measured region is larger than that in the simulation and theoretical prediction. Although the change in the trend of the reflectivity curve in the published work [16] is similar to that in this study, the reflectivity near the edge of the measured region is extremely large. This is because the size of the prepared dendritic structure deviates from that of the design, and the prepared Ag film does not completely reflect the incident light. Therefore, the position-dependent reflectivity of the dendritic metasurface sample is in fact a statistical result, and its performance is consequently reduced compared with that of the periodic metasurface [30].

## 4. Conclusions

Ag dendritic metasurfaces that can perform integral operation in the yellow and red bands were designed in this study. The position-dependent reflection coefficients obtained by simulation were consistent with the theoretical predictions. The position-dependent reflectivity of the prepared Ag dendritic metasurface sample was measured in the red band. The change in the trend of the reflectivity curve substantially agreed with those of the simulation and theoretical prediction. This dendritic metasurface, which can perform mathematical operations in the visible bands, provides the possibility of miniaturized and integrated all-optical signal processing systems. Furthermore, its integral property can be used for real-time signal processing and communication, beam shaping, and in several other fields. The metasurface sample was prepared by the bottom-up electrochemical deposition method, whose process is simple and mature, and involves low costs, making it highly practical.
